# Immunoinformatics-driven design of a conserved RNA-dependent RNA polymerase-based multi-epitope vaccine against avian infectious bronchitis virus

**DOI:** 10.14202/vetworld.2026.448-468

**Published:** 2026-01-31

**Authors:** Reza Rezaei, Gholamreza Nikbakht Brujeni, Bahman Abedi Kiasari, Fateme Frootan, Mohammad Hossein Mokhtarian, Salar Golabdar

**Affiliations:** 1 Department of Microbiology and Immunology, Faculty of Veterinary Medicine, University of Tehran, Tehran, Iran; 2 Maya Milk, Inc, Delaware, USA; 3 Department of Viral Diseases, Sana Institute for Avian Health and Diseases Research, Tehran, Iran

**Keywords:** avian coronavirus, avian infectious bronchitis virus, immunoinformatic, multi-epitope vaccine, RNA-dependent RNA polymerase, toll-like receptor 7, vaccine design

## Abstract

**Background and Aim:**

Avian infectious bronchitis virus (IBV) is a highly contagious coronavirus that causes severe respiratory, renal, and reproductive disease in chickens, resulting in significant economic losses in the poultry industry worldwide. The high mutation and recombination rates of IBV, especially in structural proteins like the spike glycoprotein, limit the effectiveness of current live attenuated and inactivated vaccines. This study aimed to design and computationally evaluate a novel multi-epitope vaccine (MEV) targeting the highly conserved RNA-dependent RNA polymerase (RdRp) of IBV in order to provide broad and lasting immune protection.

**Materials and Methods:**

The RdRp protein (NCBI: NP_740629.1) was chosen as the vaccine target due to its high sequence conservation and crucial role in viral replication. B-cell lymphocyte, cytotoxic T-lymphocyte, and helper T-lymphocyte epitopes were predicted using various immunoinformatics tools, followed by strict screening for antigenicity, non-allergenicity, non-toxicity, interferon-γ induction potential, and lack of homology with *Gallus gallus* proteins. The selected epitopes were assembled into a single construct with suitable linkers, incorporating avian β-defensin 8 as an N-terminal adjuvant. The vaccine candidate was analyzed in *silico* for physicochemical properties, structural stability, solubility, molecular docking with chicken Toll-like receptor 7 (TLR7), molecular dynamics, and immune response simulation.

**Results:**

The final multi-epitope construct showed favorable physicochemical properties, including high stability (instability index: 25.74), hydrophilicity, and predicted solubility (Protein-Sol score: 0.504). Structural modeling and validation confirmed a reliable tertiary structure. Molecular docking demonstrated strong, stable binding to TLR7, supported by multiple hydrogen bonds and salt bridges, while molecular dynamics analysis indicated sufficient flexibility for immune recognition. Immune simulations forecasted robust humoral and cellular immune responses, characterized by increased IgG levels, expansion of memory B and T cells, and a Th1-biased cytokine profile with significant interferon-γ production.

**Conclusion:**

This immunoinformatics-designed RdRp-based MEV is a promising candidate for broad-spectrum protection against IBV. By targeting a conserved non-structural protein, it may address limitations linked to strain-specific vaccines. *In vitro* and *in vivo* testing is needed to confirm its safety, immunogenicity, and protective efficacy in poultry.

## INTRODUCTION

Avian infectious bronchitis (IB) is a highly contagious viral disease that impacts commercial poultry production worldwide and causes significant economic losses. Because of its major global impact, IB is regularly included in the annual reports of the World Organization for Animal Health, and extensive research over the past fifty years has focused on understanding the disease and reducing its effects [1]. The disease is caused by IB virus (IBV), an envelope virus with a positive-sense, single-stranded RNA (ssRNA) genome that belongs to the genus *Gammacoronavirus* within the family *Coronaviridae* and the order *Nidovirales*. Chickens of all ages are natural hosts of IBV. Although the virus mainly targets the upper respiratory tract, it can also infect the gastrointestinal, reproductive, and urinary systems [2, 3], leading to serious economic losses related to nephropathogenesis and impaired fertility. In broiler chickens, IBV infection often results in decreased weight gain and mortality rates of 10% to 60% during the first 4 to 6 weeks of life. In laying hens, long-term effects include a 20%–70% decrease in egg production and deterioration in egg quality, negatively affecting hatchability and market value [3, 4].

A major challenge in IB control is the high mutation and recombination rates of IBV, which drive the ongoing emergence of new strains with distinct antigenic profiles, enabling immune escape. This antigenic diversity greatly reduces vaccine effectiveness, as immunity from one serotype may not provide sufficient protection against different strains [5, 6]. Consequently, IB remains a persistent and unresolved issue in poultry health management, causing significant financial losses worldwide despite widespread vaccination efforts [7].

Over recent decades, IB control strategies have primarily relied on live attenuated and inactivated IBV vaccines. However, live attenuated vaccines pose a risk of reverting to virulence or recombining with circulating field strains, thereby contributing to the emergence of new IBV variants [2, 8]. Although inactivated vaccines are safer, their limited ability to induce strong T-cell-mediated immune responses reduces their overall protective effectiveness. Still, vaccination remains the most effective method currently available for IB control [2], highlighting the urgent need to develop safer and more effective IBV vaccines.

The IBV genome consists of approximately 27.6 kb of single-stranded, positive-sense RNA and is organized into two main regions: polymerase genes and structural/accessory genes [3]. The polymerase region, which makes up the first two-thirds of the genome, encodes two overlapping open reading frames (ORF), ORF1a and ORF1b. The remaining third encodes four structural proteins: envelope (E), spike (S), membrane (M), and nucleocapsid (N) [3, 4]. Translation of ORF1a and ORF1b produces two large polyproteins, Polyprotein (pp)1a and pp1ab, which are cleaved after translation to produce non-structural proteins (NSPs) [3]. These NSPs, including nsp12 or RNA-dependent RNA polymerase (RdRp), are vital for coronavirus RNA replication and transcription.

Given the significant antigenic variability of structural proteins, such as the S glycoprotein, targeting conserved NSPs offers a promising alternative vaccine approach. RdRp is a highly conserved enzyme responsible for viral RNA synthesis and has no homologs in host cells, making it an attractive target for peptide-based vaccine development. Its unique structural features, including domains beyond the catalytic core, distinguish it from other viral RdRps and create opportunities for selective immune targeting. This high degree of conservation makes RdRp an ideal candidate for multi-epitope vaccine (MEV) design, with the potential to provide broad protection against various IBV strains despite ongoing antigenic variation [3, 9, 10].

Accordingly, this study emphasizes RdRp as a highly conserved and critical viral target to reduce immune escape. MEV strategies are an innovative approach for IBV control because they can activate both B-cell- and T-cell-mediated immune responses by including conserved immunogenic epitopes [11]. Cytotoxic T-lymphocyte (CTL) responses are essential for destroying virus-infected cells, while neutralizing antibodies are key in eliminating circulating virus particles [12, 13]. By combining B-cell, CTL, and helper T-lymphocyte (HTL) epitopes into a single construct, MEVs provide a broad immunization strategy and reduce the risks of allergenicity and toxicity [14].

Epitope-based vaccines designed through *in silico* approaches offer several advantages over traditional vaccine development methods. These strategies eliminate the need for *in vitro* cultivation of pathogenic microorganisms, thereby improving biological safety. Additionally, they enable the rational selection and combination of epitopes from conserved viral regions, which promotes precise and targeted immune responses [15]. Immunoinformatics has become a powerful tool in vaccine design, providing high specificity, lower experimental costs and timelines, improved safety profiles, and the ability to predict long-lasting innate and adaptive immune responses [16]. Through computational modeling, molecular docking with innate immune receptors, and dynamic simulations, immunoinformatics enables systematic validation of vaccine constructs for structural stability and binding affinity [17].

*In silico* methods have become well-established for quickly identifying potential B-cell and T-cell epitopes, speeding up peptide-based vaccine research and development compared to traditional experimental methods. These approaches have been successfully used to find antigenic regions within NSPs of severe acute respiratory syndrome coronavirus 2 (SARS-CoV-2), including RdRp [18, 19]. Unlike earlier immunoinformatics studies in poultry that mainly focused on the highly variable S protein to achieve serotype-specific immunity [20, 21], or combined structural and NSPs to expand coverage at the cost of increased complexity [22, 23], this study takes a targeted approach focused solely on the conserved RdRp protein.

Despite decades of extensive research and widespread vaccination efforts, avian IB remains a significant challenge to global poultry health due to the rapid genetic changes and antigenic variation of the IBV. Most current vaccines and immunoinformatics-based vaccine design studies primarily target structural proteins, especially the S glycoprotein, because of its role in virus attachment and neutralization. However, the high mutation and recombination rates in the S gene often result in immunity specific to certain serotypes and fail to provide sufficient cross-protection against new IBV variants. As a result, vaccine effectiveness is often short-term and requires regular updates, contributing to ongoing outbreaks despite widespread vaccination.

Although NSPs are crucial for viral replication and transcription and are generally more conserved across IBV lineages, they remain underexplored as vaccine targets. Specifically, the RdRp, nsp12, an enzyme vital to replication that lacks host homologs, has received limited attention in IBV vaccine research. So far, few studies have systematically assessed RdRp-derived epitopes for their ability to trigger both humoral and cellular immune responses in poultry. Additionally, current immunoinformatics studies on avian viruses often rely on either single epitope classes or mixed protein constructs, which increase structural complexity and may lead to unintended junctional immunogenicity.

Another crucial gap exists in the limited integration of innate immune receptor engagement, structural validation, molecular dynamics, and immune simulation into IBV vaccine development pipelines. Many computational studies halt at epitope prediction without evaluating receptor-binding, conformational stability, solubility, or long-term immune memory. Additionally, the lack of avian-specific major histocompatibility complex (MHC) datasets has limited the rational selection of T-cell epitopes, leading to inconsistent or incomplete assessments of cellular immune responses. Together, these limitations underscore the need for a targeted, structure-guided, and immunologically comprehensive vaccine design approach focused on conserved IBV proteins that elicit durable, cross-protective immunity.

This study aimed to fill these gaps by using an integrated immunoinformatics approach to design and assess a new MEV targeting the highly conserved RdRp of IBV. Specifically, it focused on identifying and prioritizing conserved B-cell, cytotoxic T-lymphocyte (CTL), and helper T-lymphocyte (HTL) epitopes from the RdRp protein through multiple predictive algorithms, followed by careful screening for antigenicity, non-allergenicity, non-toxicity, interferon-γ induction potential, and absence of homology with *Gallus gallus* proteins.

Furthermore, the study aimed to design a rational MEV that includes an avian-specific immunostimulatory adjuvant and optimized peptide linkers to improve antigen processing and immune activation. Extensive in *silico* analyses were conducted to assess the physicochemical properties, structural integrity, solubility, and stability of the vaccine construct. The interaction between the designed vaccine and chicken Toll-like receptor 7 (TLR7) was further examined through molecular docking and molecular dynamics simulations to explore innate immune engagement. Lastly, immune response simulations were carried out to forecast the strength, quality, and durability of humoral and cellular immune responses.

By focusing solely on a conserved NSP crucial for viral replication, this study aimed to establish a proof-of-concept framework for developing a broadly protective, safe, and scalable IBV vaccine candidate, thus contributing to next-generation vaccine strategies against rapidly evolving avian coronaviruses.

## MATERIALS AND METHODS

### Ethical approval

This study was conducted entirely using *in silico* immunoinformatics approaches and did not involve the use of live animals, animal-derived tissues, clinical samples, or human participants at any stage of the research. As per institutional guidelines, formal ethical approval was not required for the computational analyses performed in this study. All data used were retrieved from publicly accessible databases, including the National Center for Biotechnology Information (NCBI), Immune Epitope Database (IEDB), and other open-access bioinformatics resources, in accordance with their respective data usage policies.

### Study period and location

This *in silico* study was conducted between January 2024 and June 2025 at the Department of Microbiology and Immunology, Faculty of Veterinary Medicine, University of Tehran, Tehran, Iran.

### Methodological workflow for the design of MEVs

**Figure 1:**
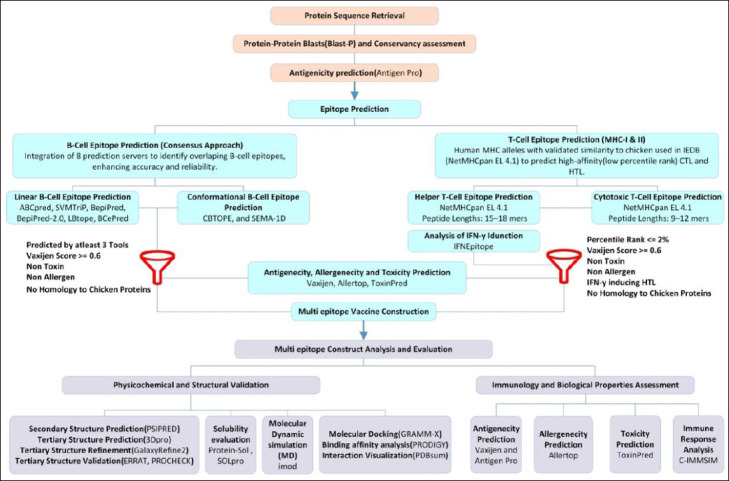
Workflow diagram illustrating the design process of a candidate multi-epitope vaccine for infectious bronchitis virus.

Figure 1 outlines the overall workflow for designing the MEV candidate against avian IBV. This graphical flowchart demonstrates the step-by-step methodology used in this in *silico* study.

### Target protein retrieval and antigenicity assessment

The RdRp protein of IBV was selected as the primary target for designing a MEV. The RdRp protein sequence from the Beaudette strain (NCBI accession number: NP_740629.1), consisting of 940 amino acids, was solely retrieved from the NCBI protein database (https://www.ncbi.nlm.nih.gov/protein/) for this in *silico* proof-of-concept study. To assess the representativeness and conserved nature of this specific Beaudette RdRp sequence, a preliminary protein-protein Basic Local Alignment Search Tool (blastp) search was conducted against the NCBI non-redundant protein sequence database (https://blast.ncbi.nlm.nih.gov/Blast.cgi?PAGE=Proteins), setting the organism to IBV (taxid:11120) with a maximum of 5000 target sequences. Afterwards, the intrinsic antigenic potential of the selected RdRp sequence was evaluated using ANTIGENpro (http://scratch.proteomics.ics.uci. edu/) [24], a sequence-based antigenicity prediction tool available on the SCRATCH Protein Predictor server.

### B-cell lymphocyte (BCL) epitope prediction

The amino acid sequence of the RdRp protein was analyzed using a combination of 8 tools to predict BCL epitopes with high confidence. An amino acid sequence predicted as an epitope by multiple independent tools has a high likelihood of being a true epitope. We improve the reliability of epitope detection by using multiple prediction tools trained on different datasets and based on various algorithms [25]. All tools were used with their default thresholds and settings. Supplementary File 1 provides detailed information about the tools used in this study, including both linear and conformational BCL prediction tools. These tools, include ABCpred, SVMTriP, BepiPred, BepiPred-2.0, LBtope, BCePred, CBTOPE, and SEMA-1D.

#### Prediction of linear BCL epitope

ABCpred (https://webs.iiitd.edu.in/raghava/abcpred/) [26, 27]; SVMTriP (http://sysbio.unl.edu/ SVMTriP) [28]; Bepipred [29] and Bepipred-2.0 [30], from the IEDB (IEDB: http://tools.iedb.org/main/tools-api/); LBtope (https://webs.iiitd.edu.in/raghava/lbtope/) [24]; and BCePred (https://webs.iiitd.edu.in/raghava/bcepred/ bcepred_submission.html) [31] were used to predict linear BCL epitopes based on properties such as hydrophilicity, flexibility, polarity, and exposed surface.

#### Prediction of conformational BCL epitope

CBTOPE (https://webs.iiitd.edu.in/raghava/cbtope/) [32, 33] and SEMA-1D (https://sema.airi.net/) [34] were used to predict conformational epitopes.

#### BCL epitopes selection criteria

BCL epitopes were chosen based on a combination of physicochemical and predictive criteria to ensure accuracy and biological importance. Eligible epitopes met these standards: (i) a sequence length between 6 and 50 amino acids, aligning with the input requirements of the screening tools, specifically, a minimum length of ≥ 6 amino acids for antigenicity prediction in VaxiJen and a maximum length of ≤ 50 amino acids for toxicity analysis in ToxinPred; and (ii) prediction by at least three separate computational tools, with identification as overlapping regions across various platforms to ensure consensus and improve prediction reliability.

### T-cell epitope prediction

We used human MHC alleles as functional surrogates for T-cell epitope prediction because of the limited availability of well-characterized chicken MHC allele data in immunoinformatics tools. Several studies have shown a significant degree of structural and functional homology between Human leukocyte antigen (HLA) molecules and the chicken MHC, especially in antigen processing and presentation pathways [35–37]. In particular, chicken MHC class I (B–F) alleles have demonstrated antigen-presentation capabilities similar to those of human class I molecules [38, 39]. Based on these findings, human alleles most closely associated with avian immune responses were selected following previous research recommendations [40, 41].

### MHC class I (CTL) epitope prediction

For CTL epitope prediction, the human class I alleles HLA-B*41:03, HLA-B*41:04, and HLA-B*40:06 were chosen as proxies for avian class I MHC alleles. Epitope prediction was performed using the IEDB analysis resource (http://tools.iedb.org/mhci/) with the IEDB-recommended method, which automatically selects the best prediction algorithm for each allele. The NetMHCpan EL 4.1 algorithm (https://tools.iedb.org/mhci/help/), which integrates binding affinity and antigen processing efficiency, was used to predict naturally processed and presented peptides [42]. CTL epitopes ranging from 9 to 12 amino acids in length were evaluated, and only those with a percentile rank below 2%, indicating high binding affinity, were chosen for further analysis.

### MHC class II (HTL) epitope prediction

For HTL epitope identification, the human MHC class II alleles HLA-DRB1*14:82, HLA-DRB1*13:66, HLA-DRB1*13:10, and HLA-DRB1*14:45 were selected as substitutes for avian class II alleles. Prediction was carried out using the IEDB Class II epitope analysis tool (http://tools.iedb.org/mhcii/), following the IEDB-recommended approach that defaults to NetMHCIIpan EL 4.1 (https://tools.iedb.org/mhci/help/) [42]. This method combines peptide-binding potential with the likelihood of natural presentation. Peptides ranging from 15–18 residues were evaluated, and those with predicted top-binding affinities (percentile rank <2%) were kept for further analysis. This threshold is a common default in immunoinformatics tools (e.g., NetMHCpan) that indicate high-affinity binders and help identify potential T-cell epitopes accurately.

### Epitope selection workflow and screening parameters

To ensure the selection of high-quality candidates for inclusion in the vaccine construct, we subjected the predicted epitopes to a series of stringent screening criteria. Each epitope was required to demonstrate (i) sufficient antigenicity (VaxiJen score ≥ 0.6), (ii) non-allergenicity, (iii) non-toxicity, (iv) potential to induce interferon-gamma (IFN-γ) responses in the case of helper T-cell (HTL) epitopes, and (v) absence of significant homology with chicken host proteins.

The antigenicity of the predicted epitopes was evaluated using the VaxiJen v2.0 server (http://www.ddg-pharmfac.net/vaxijen/) [43], which classifies antigens based on the physicochemical properties of proteins without relying on sequence alignment. An initial antigenicity prediction was performed with the default threshold of 0.4. For final epitope selection in the multi-epitope construct, a more rigorous cut-off of ≥0.6 was used to include only epitopes with the highest predicted antigenicity, thereby enhancing the confidence and specificity of the selected candidates.

Epitope allergenicity was evaluated using AllerTop (default settings) (https://www.ddg-pharmfac.net/ AllerTOP) [44]. In this tool, amino acid properties are analyzed based on five indices: 1. molecular size, 2. amino acid hydrophobicity, 3. tendency to form alpha-helices, 4. relative frequency of amino acids, and 5. propensity to form beta-sheets. The server assesses allergenicity through cross-covariance. Combining these indices can serve as a predictor of the sequence’s three-dimensional structure and provide a more accurate estimate of its allergenic potential.

The toxicity and IFN-induction were assessed using Toxinpred (http://crdd.osdd.net/raghava/ toxinpred) [45, 46] and IFNepitope (https://webs.iiitd.edu.in/raghava/ifnepitope) [47], respectively. All analyses employed default settings to ensure reproducibility. The final epitopes chosen for inclusion in the construct were checked for sequence similarity against the *Gallus gallus* proteome to reduce the risk of autoimmune responses and cross-reactivity. Protein-protein similarity searches were conducted using the BLASTp algorithm (NCBI BLAST; taxid:9031) through the Protein BLAST interface (https://blast.ncbi.nlm.nih.gov/Blast.cgi?PAGE=Proteins). Each final epitope was individually queried, and sequences with significant homology to chicken proteins (E-value ≤ 0.05) were excluded from further analysis. Epitopes with E-values>0.05 were deemed nonhomologous and retained for incorporation into the MEV construct.

### Construction of MEV

To develop a MEV against IBV, selected CTL, HTL, and BCL epitopes were linked together into a single polypeptide using carefully chosen peptide linkers. CTL epitopes were connected with the Ala-Ala-Tyr (AAY) linker, which supports proteasomal cleavage and enhances MHC class I epitope presentation, thereby promoting effective cytotoxic T-cell responses. HTL epitopes were joined with Gly-Pro-Gly-Pro-Gly (GPGPG) linkers to preserve conformational flexibility and reduce the formation of junctional or chimeric epitopes. These linkers also facilitate CD4+ T-cell activation, which is vital for a strong and lasting immune response. BCL epitopes were separated by Lys-Lys (KK) linkers to prevent the creation of artificial epitopes at junctions and to reduce the risk of unintended antibody production against linker-epitope fusions [48]. These linkers play important structural and immunological roles by maintaining epitope integrity and supporting proper folding and processing [49].

Avian β-defensin 8 (NCBI accession number: NP_001001781.1) was included at the N-terminal of the vaccine construct as an immune-stimulating adjuvant and linked to the epitope assembly using an Glu-Ala-Ala-Ala-Lys (EAAAK) linker, selected for its rigid α-helical structure that promotes functional separation between domains and enhances the overall stability of the vaccine construct [50]. This adjuvant was chosen because it is endogenously expressed in chickens, where it acts as a powerful immunomodulator by activating TLR pathways, including TLR7, and inducing proinflammatory cytokines such as IFN-γ and interleukin (IL)-6 in avian immune cells, aligning with the vaccine’s focus on TLR7-mediated innate immunity against ssRNA viruses like IBV [35, 50]. In comparison, alternative adjuvants like flagellin (a bacterial TLR5 agonist) or CpG motifs (TLR9 agonists) were not prioritized because they are exogenous and could pose risks of non-specific inflammation or integration issues in peptide-based constructs, whereas β-defensin 8 provides avian-specific compatibility and seamless fusion for improved safety and efficacy in chicken models [15]. The use of these carefully selected linkers and adjuvant elements ensures optimal spatial arrangement, supports proper antigen processing pathways, and fosters a broad-based immune response [49].

### Evaluation of the physicochemical and safety properties of the vaccine

### Analysis of physicochemical properties

The physicochemical properties of the MEV were assessed using the Expasy ProtParam server (https://web.expasy.org/protparam/) [51].

### Antigenicity, allergenicity, and toxicity assessment

The vaccine’s antigenicity was predicted with the ANTIGENpro [26] server and VaxiJen [43]. The allergenicity and toxicity of the vaccine were assessed using the AllerTop [44] and ToxinPred [45, 46] servers, respectively.

### Structural modeling, refinement, and validation

### Prediction of secondary structures

The MEV was analyzed with the PSIPRED 4.0 server (http://bioinf.cs.ucl.ac.uk/psipred/) to examine the secondary structure [52].

### Tertiary structure modeling and refinement

The 3D structure was generated using the SCRATCH 3Dpro server [53] (https://scratch.proteomics.ics. uci.edu/) and refined with the GalaxyRefine2 web server (http://galaxy.seoklab.org/) [54].

### Structural validation

ERRAT (https://saves.mbi.ucla.edu/) [55] and PROCHECK (https://saves.mbi.ucla.edu/) [56] from the SAVES server were used for model validation, and the ProsA server (https://prosa.services.came.sbg.ac.at/prosa.php) [57] was used to analyze protein structure errors.

### Solubility prediction

We assessed the solubility of the vaccine construct using two computational tools: Protein-Sol (https://protein-sol.manchester.ac.uk) [58], which predicts solubility based on algorithm-driven analysis of sequence properties, and SOLpro (http://scratch.proteomics.ics.uci.edu) [59], a sequence-based predictor of protein solubility upon expression. Protein-Sol compares the vaccine’s solubility score against a benchmark derived from *Escherichia coli* proteins, while SOLpro evaluates solubility using a probability threshold. Both tools were used with default settings to ensure reproducibility and consistency in predicting the potential for recombinant production of the construct.

The Protein-Sol threshold of 0.45, established as the average solubility score for *E. coli* proteins, serves as a key benchmark for determining whether a recombinant protein can be efficiently expressed and purified, a crucial factor for scalable vaccine production. A score above this threshold indicates less aggregation and better processability, improving the vaccine’s feasibility for poultry immunization. The SOLpro threshold of 0.5 distinguishes soluble (≥0.5) from insoluble (<0.5) proteins, providing a standardized way to evaluate the success of SOLpro expression. These thresholds are biologically important because they help ensure the vaccine remains stable and bioavailable, supporting its immunogenicity and practical use against IBV in chickens.

### Molecular docking analysis

To improve our IBV vaccine design, we examined how the vaccine construct binds to chicken TLR7 (UniProt ID: C4PCM1, AlphaFold 3D structure: https://alphafold.ebi.ac.uk/entry/C4PCM1), a key receptor for sensing viruses in poultry. TLR7 was chosen because of its known role in detecting ssRNA viruses, such as IBV. It is crucial for triggering immune responses against IBV by recognizing viral RNA and promoting cytokine production, along with TLR3 and MDA5. Strong immune activation depends on effective antigen–receptor interactions, which are vital for pathogen detection. Therefore, we docked the vaccine with TLR7, one of 10 avian TLR genes [60, 61], using the GRAMM-X server (http://vakser.compbio.ku.edu/resources/gramm/grammx/) [62, 63] to simulate their interaction. The 3D structure of chicken TLR7 (UniProt ID: C4PCM1) was sourced from the AlphaFold Protein Structure Database (https://alphafold.ebi.ac.uk/), as no experimentally determined crystal structure is currently available in the Protein Data Bank (PDB). The binding affinities of the top docking clusters were estimated at 25°C using the PRODIGY server (https://wenmr.science.uu.nl/prodigy/) [64]. The vaccine–TLR7 interface was visualized using PDBsum (https://www.ebi.ac.uk/thorntonsrv/databases/pdbsum/Generate.html) [65] and PyMOL (https://pymol.org/2/) [66], revealing a stable interaction that may enhance immunogenicity.

### Molecular dynamics simulation

We used the iMODS server (https://chaconlab.org/multiscale-simulations/imod) [67, 68] for normal mode analysis (NMA) in internal coordinates to assess the stability and dynamic behavior of the IBV vaccine construct. This method examined collective atomic movements within the vaccine model, offering insights into its structural integrity for effective poultry vaccine development. iMODS produced key analyses, including deformability, which measures the flexibility of the protein at hinge regions and indicates its ability to adapt during antigen processing and immune recognition. The eigenvalue, representing the energy needed to deform the structure, was calculated to evaluate molecular stiffness; a lower eigenvalue indicates greater flexibility, enhancing the vaccine’s conformational adaptability for stable presentation to immune receptors. Other outputs included B-factors, variance and covariance matrices, and elastic network models, which collectively assess residue displacement and stability, critical factors for maintaining the vaccine’s immunogenicity during production and immune interaction [67, 68].

### Immune simulation and response prediction

We conducted computational immune simulations using the C-ImmSim server (https://kraken.iac.rm. cnr.it/C-IMMSIM/) to evaluate the immune response triggered by the MEV [69]. C-ImmSim is an agent-based computational model that simulates humoral and cellular immune responses by modeling interactions among antigens, antibodies, and immune cells within a virtual lymphoid environment. Each immune component is represented as a bit string, and the simulator tracks processes such as antigen recognition, clonal expansion, affinity maturation, and memory cell formation over time. The 1,100 time steps simulate roughly one year to capture long-term memory, with each step representing 8 h. Three doses were administered at steps 1, 84, and 168 (days 0, 28, and 56), following standard prime–boost–boost protocols in C-ImmSim for evaluating Th1-biased responses in vaccines. This dosing schedule aligns with the protocol described by Castiglione et al. [70], with a minimum 28-day interval between doses, and matches common IBV boost schedules in broilers and layers to facilitate adaptive immunity development [71]. We included the MHC alleles A0101 (used twice) for MHC class I (A), B4006 and B4103 for MHC class I (B), and DRB1_1310 and DRB1_1445 for MHC class II (DR). These alleles were chosen based on their association with predicted T-cell epitopes or as default options recommended by the server when epitope-specific data were unavailable. C-ImmSim-generated cytokine and antibody profiles, including increased IFN-γ and IL-2, which indicate a Th1-biased response, provide insights into the strength and type (Th1/Th2) of the immune response, enabling assessment of the vaccine’s potential immunogenicity against IBV [69].

## RESULTS

### Sequence acquisition of target protein and antigenicity assessment

The retrieved RdRp protein sequence from the IBV Beaudette strain (NCBI accession number: NP_740629.1) is 940 amino acids long. A preliminary protein-protein BLAST (blastp) search against the NCBI non-redundant protein database yielded 939 results to assess its representativeness and conserved nature. Of these, 932 sequences (about 99.25%) showed over 90% identity to the Beaudette RdRp, with 815 sequences demonstrating over 95% identity. This high level of conservation and broad homology across many IBV isolates highlight the Beaudette RdRp sequence as a highly representative and reliable model for this in *silico* study. Later, its inherent antigenicity was evaluated. ANTIGENpro predicted the RdRp protein as a probable antigen, with an antigenicity score of 0.572833. This score, which surpasses typical thresholds for antigenic proteins, indicates a strong potential to induce an immune response and supports its selection for subsequent epitope prediction.

### Prediction and selection of BCL epitopes

After reviewing all epitopes provided by the tools, consensus sequences predicted to be epitopes in at least three (out of eight) tools were selected. The chosen epitopes were then screened based on the following criteria: no allergenicity, non-toxicity, and Vaxijen antigenicity score of at least 0.6. They were subsequently ranked by antigenicity score, and the top five epitopes were selected for construction of a MEV. The highest-scoring BCL epitope on VaxiJen received a score of 1.271. The BCL epitopes listed in Table 1 were identified using multiple prediction tools. Some epitopes also overlap with CTL epitopes, indicating their potential to stimulate both arms of the adaptive immune system. All predicted BCL epitopes, including those not selected, are provided in Supplementary Table 1.

**Table 1:** Non-allergenic and non-toxic antigenic B cell epitopes along with their respective antigenicity scores were determined. The longest sequence is at the top of the final column in bold black. In remaining sequences, shared regions are bold, while non-shared regions are in regular text.

Consensus sequence	Location	Length	Vaxijen score	Nested epitopes prediction method
YRRVNFDPAFVEKFYS				**YRRVNFDPAFVEKFYS (ABCPred)**
	740-755	16	1.271	YRR**VNFDPAFV**EKFYS (Bepipred2)
				Y**RRVNFDPA**FVEKFYS (LBtope)
				**LVEVDGEPKYLPYPDP (ABCPred)**
				**LVEVDGEPKYLPYPDP** (CBTope)
LVEVDGEPKYLPYPDP	827-842	16	0.7709	L**VEVDGEP**KYLPYPDP (SEMA-1D)
				LVEVD**GEPKYLPY**PDP (BcePred_Accessibility)
				L**VEVDGEPKYLP**YPDP (CTL epitope/NetMHCpan EL)
				LVEVD**GEPKYLPYP**DP (CTL epitope/NetMHCpan EL)
GIYVKPGGTSSGDATT				**GIYVKPGGTSSGDATT (ABCPred)**
	680-695	16	0.7662	GIYV**KPGGTSSGDAT**T (Bepipred2)
				GIYV**KPGGTS**SGDATT (BcePred_Flexibility)
				GIYVKPG**GTSSGDA**TT (BcePred_Hydrophilicity)
				**HEKSCYEDLKSEVTADHDFF (SVMTrip)**
HEKSCYEDLKSEVTADHDFF	91-110	20	0.6052	HEK**SCYEDLKSEVTADHDF**F (ABCPred)
				HEKSCYEDL**KSEVTA**DHDFF (Bepipred)
				HEKSCYEDL**KSEVTADHDF**F (CTL epitope/NetMHCpan EL)
				**YVKPGGTSSGDATTAY (Bepipred)**
YVKPGGTSSGDATTAY	682-697	16	0.6008	YV**KPGGTSSGDAT**TAY (Bepipred2)
				YV**KPGGTS**SGDATTAY (BcePred_Flexibility)
				YVKPG**GTSSGDA**TTAY (BcePred_Hydrophilicity)

### Prediction and selection of T-cell epitopes

High-affinity MHC class I and II epitopes (percentile ranks ≤2%) were selected for further analysis. Predicted epitopes were filtered based on their antigenicity, allergenicity, and toxigenicity, as well as their capacity to induce IFN-γ production for MHC class II. Three epitopes from each MHC class, with the highest antigenicity, and lacking allergenicity and toxicity, were chosen.

### Helper T-cell (HTL) epitopes

Three HTL epitopes were chosen from key regions of the RdRp protein based on MHC class II binding predictions. Epitopes overlapping adjacent areas were selected to ensure the best MHC II presentation. Notably, two CTL epitopes were embedded within the HTL sequences, potentially enabling the simultaneous activation of helper and cytotoxic T cells (Table 2). Supplementary Table 2 offers additional details on all predicted HTL epitopes in this study, including those not used in the final vaccine construct.

**Table 2:** Non-allergenic and non-toxic antigenic HTL epitopes were selected for multi-epitope vaccine construction along with their antigenicity scores. All epitopes demonstrated positive in silico IFN-γ induction, as predicted by the IFNepitope server. The consensus sequence is at the top of the final column in bold black. In remaining sequences, shared regions are bold, while non-shared regions are in regular text.

Consensus sequence	Location	Length	Vaxijen score	Alleles	Overlapping epitopes
SLEEQDQLFEITKKNVLPT				HLA-DRB1*14:82	**SLEEQDQLFEITKKNVLPT**
	528-546	19	0.9948	HLA-DRB1*13:10	**SLEEQDQLFEITKKNVLP**T
				HLA-DRB1*14:45	S**LEEQDQLFEITKKNVLP**T
				HLA-DRB1*13:66	S**LEEQDQLFEITKKNVLPT**
				HLA-DRB1*14:82	**QDQLFEITKKNVLPTITQM**
QDQLFEITKKNVLPTITQM	532-550	19	0.7745	HLA-DRB1*13:10	**QDQLFEITKKNVLPTIT**QM
				HLA-DRB1*14:45	**QDQLFEITKKNVLPTITQ**M
				HLA-DRB1*13:66	Q**DQLFEITKKNVLPTITQ**M Q**DQLFEITKKNVLPTITQM**
				HLA-DRB1*13:10	**NLKYAISAKNRARTV**

### Cytotoxic T-cell (CTL) epitopes

A total of five CTL epitopes were nested within either B-cell or HTL epitopes, indicating their potential as multifunctional epitopes capable of engaging both cellular and humoral immune responses. The CTL epitope 100 KSEVTADHDF 109 is fully nested within the BCL epitope 100 HEKSCYEDLKSEVTADHDFF 118. Similarly, 528 SLEEQDQLFEI 538 and 534 QLFEITKKNVL 544 are embedded within HTL epitopes 528 SLEEQDQLFEITKKNVLPT 545 and 530 QDQLFEITKKNVLPTITQM 548, respectively. The CTL epitopes 828 VEVDGEPKYLP 838 and 832 GEPKYLPYP 840 are both nested within the BCL epitope 827 LVEVDGEPKYLPYPDP 842. These overlapping regions highlight the immunological importance of these epitopes and support their potential use in MEV design to elicit a comprehensive immune response. Additionally, three top CTL epitopes with the highest VaxiJen scores were embedded in the vaccine construct to further boost CD8+ T-cell responses (Table 3). Predicted CTL epitopes that nested within the BCL and HTL epitopes are presented in Table 4, and a complete list of all predicted CTL epitopes, including nonselected candidates, is provided in Supplementary Table 3.

**Table 3:** Non-allergenic and non-toxic antigenic CTL epitopes were selected for multi-epitope vaccine construction along with their antigenicity scores and percentile ranks.

Consensus sequence	Alleles	Percentile Rank	Location	Length	Vaxijen Score
TQMNLKYAI	HLA-B*41:04	1.3	548-556	9	1.2979
	HLA-B*40:06	1.3			
VEKGYVGVITL	HLA-B*41:04	0.18	206-216	11	0.8833
	HLA-B*41:03	0.39			
	HLA-B*40:06	0.96			
TEDGNLEYL	HLA-B*41:03	0.09	67-75	9	1.0216
	HLA-B*41:04	0.5			
	HLA-B*40:06	0.82			

**Table 4:** Predicted CTL epitopes nested within the BCL and HTL epitopes. Each CTL epitope is listed along with its location, length, VaxiJen score, and the corresponding BCL or HTL epitope in which it is nested. This overlap suggests a potential synergy between cellular and humoral immune responses. CTL epitope sequences are shown in bold black, while non-overlapping regions of larger epitopes appear in regular black text.

Predicted sequence	Location	Length	Vaxijen Score	Nested in the epitope (epitope type)
KSEVTADHDF	100-109	10	0.6712	HEKSCYEDL**KSEVTADHDF**F (BCl Epitope)
SLEEQDQLFEI	528-538	11	0.947	**SLEEQDQLFEI**TKKNVLPT (HTL epitope)
QLFEITKKNVL	534-544	11	0.6377	QD**QLFEITKKNVL**PTITQM (HTL epitope)
VEVDGEPKYLP	828-838	11	0.6671	L**VEVDGEPKYLP**YPDP (BCL epitope)
GEPKYLPYP	832-840	9	1.1675	LVEVD**GEPKYLPYP**DP (BCL epitope)

### Selection criteria and computational screening of candidate epitopes

Applying the predefined selection criteria, antigenicity (VaxiJen score ≥ 0.6), non-allergenicity, non-toxicity, IFN-γ induction potential (for HTL epitopes), and absence of *Gallus gallus* proteome homology, resulted in keeping only high-confidence epitopes for construct design. All shortlisted epitopes met the antigenicity threshold, were predicted to be non-allergenic and non-toxic, and HTL candidates showed IFN-γ-induction potential. No significant similarity (E-value > 0.05) to chicken host proteins was confirmed by BLASTp screening. These validated epitopes were then incorporated into the MEV construct. The validated epitopes were included in the MEV construct (Table 5), which summarizes the selected epitopes, including their sequences, locations, lengths, antigenicity scores, allergenicity, toxicity, epitope types (BCL, HTL, CTL), and HLA allele associations.

**Table 5:** Sequence, location, length, antigenicity (Vaxijen 2.0), allergenicity (AllerTop), toxicity (ToxinPred), epitope type (BCL, HTL, and CTL), and HLA allele associations of selected epitopes for vaccine design.

Epitope sequence	Location	Length	Antigenicity score (VaxiJen 2.0)	Allele	Allergenicity (AllerTop)	Toxicity (ToxinPred)	Epitope type
YRRVNFDPAFVEKFYS	740–755	16	1.271	—	Non-allergen	Non-toxin	BCL
LVEVDGEPKYLPYPDP	827–842	16	0.7709	—	Non-allergen	Non-toxin	BCL
GIYVKPGGTSSGDATT	680–695	16	0.7662	—	Non-allergen	Non-toxin	BCL
HEKSCYEDLKSEVTADHDFF	91–110	20	0.6052	—	Non-allergen	Non-toxin	BCL
YVKPGGTSSGDATTAY	682–697	16	0.6008	—	Non-allergen	Non-toxin	BCL
NLKYAISAKNRARTV	551–565	15	1.3422	HLA-DRB1*13:10, HLA-DRB1*13:66, HLA-DRB1*14:45	Non-allergen	Non-toxin	HTL
SLEEQDQLFEITKKNVLPT	528–546	19	0.9948	HLA-DRB1*14:82, HLA-DRB1*13:10, HLA-DRB1*14:45, HLA-DRB1*13:66	Non-allergen	Non-toxin	HTL
QDQLFEITKKNVLPTITQM	532–550	19	0.7745	HLA-DRB1*14:82, HLA-DRB1*13:10, HLA-DRB1*14:45, HLA-DRB1*13:66	Non-allergen	Non-toxin	HTL
TQMNLKYAI	548–556	9	1.2979	HLA-B*41:04, HLA-B*40:06	Non-allergen	Non-toxin	CTL
TEDGNLEYL	67–75	9	1.0216	HLA-B*41:03, HLA-B*41:04, HLA-B*40:06	Non-allergen	Non-toxin	CTL
VEKGYVGVITL	206–216	11	0.8833	HLA-B*41:03, HLA-B*41:04, HLA-B*40:06	Non-allergen	Non-toxin	CTL

### MEV development and evaluation of physicochemical and biological properties

**Figure 2:**
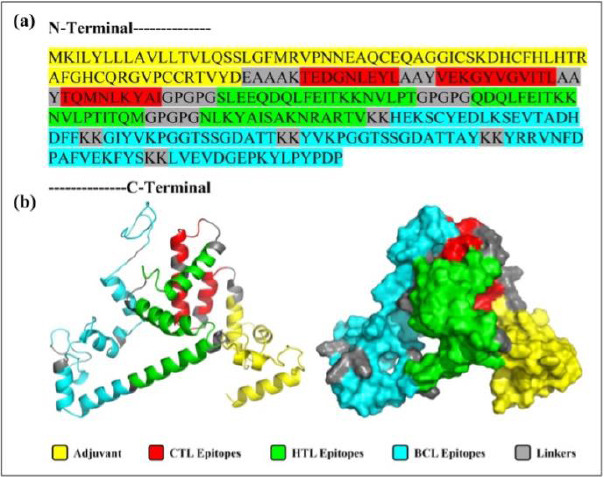
Primary Sequence and 3D visualization of the Constructed Vaccine. (a) Primary sequence of the proposed vaccine. (b) Tertiary structure predicted by the 3Dpro server, refined using Galaxy Refine2, and visualized in PyMOL in both cartoon and surface views.

The final MEV construct, comprising 268 amino acids, was assembled from selected CTL, HTL, and BCL epitopes derived from the RdRp protein of IBV. To boost immune activation, an avian β-defensin 8 sequence was added to the N-terminus as an adjuvant and linked to the epitope regions using suitable peptide linkers. Computational assessments confirmed that the designed construct is antigenic, nonallergenic, and non-toxic, indicating its safety and ability to induce a strong immune response. Figure 2 presents a schematic of the vaccine’s structure, including epitope arrangement and linker placement.

We examined the physicochemical properties of the vaccine construct using ProtParam. The results showed that the vaccine has a molecular weight of 29507.75 Daltons and an isoelectric point of 8.77, indicating its basic nature. The analysis revealed that 35 amino acids, including arginine and lysine, are positively charged, while 29 amino acids, such as glutamic acid and aspartic acid, are negatively charged. The protein is considered stable, with an instability index of 25.74, which is below the threshold of 40 for stable proteins. It is also hydrophilic, as indicated by a Grand Average of Hydropathicity (GRAVY) of −0.450. The aliphatic index of the vaccine construct was 72.05, suggesting high thermostability. Based on half-life predictions, the vaccine candidate remains stable for approximately 30 h in mammalian hosts, over 20 h in yeast, and at least 10 h in *E. coli*.

### Secondary and tertiary structure prediction, refinement, and validation

**Figure 3:**
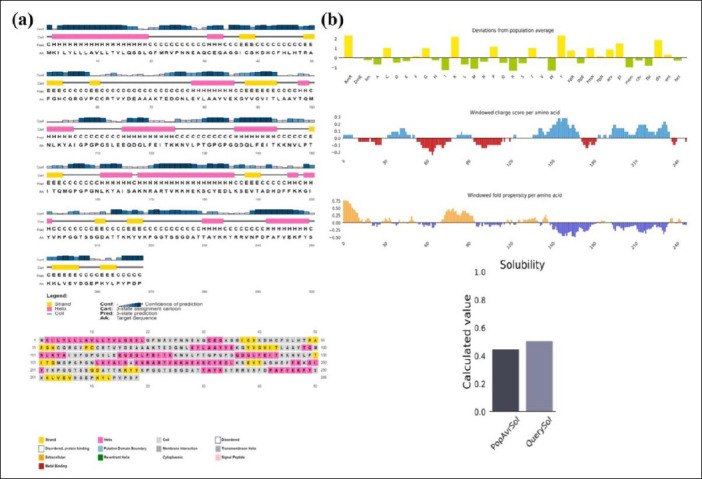
(a) Secondary structure and solubility analysis of the designed vaccine construct. Secondary structure prediction of the designed vaccine construct as analyzed by the PSIPRED server (graphical representation). The secondary structures are color-coded: α-helix (pink), β-sheet (yellow), and coil (gray). (b) Vaccine solubility compared with *E. coli* solubility

The PSIPRED 4.0 server analyzed the secondary structure of the 268-amino acid vaccine, showing that it is composed of 51.13% coil, 35.44% α-helix, and 13.43% β-strand (Figure 3). The 3Dpro tool was used to predict the protein’s tertiary structure, which was further refined with the Galaxy Refine2 tool, yielding 10 refined models.

Model 4 was chosen because of its superior structural quality, including a higher percentage of Ramachandran-favored residues (95.9%), no poor rotamers (0), a lower clash score (0.9), and a better MolProbity score (1.052). The selected model was visualized with PyMOL (Figure 3).

**Figure 4:**
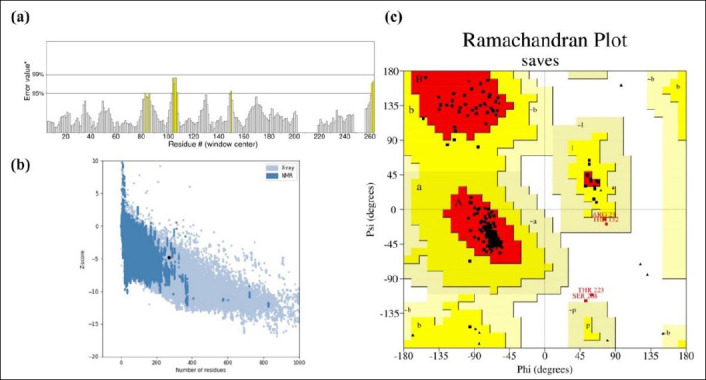
Structural Validation of the Refined Vaccine Model. (a) Validation of the predicted model’s overall quality using ERRAT analysis, with an overall quality factor of 96.1702. (b) Structural assessment using the ProSA server, with a Z-score of −4.84. (c) Ramachandran plot analysis of the designed vaccine model using the PROCHECK server, showing 92.9%, 5.3%, 0.9%, and 0.9% of residues in the favored, allowed, outlier, and disallowed regions, respectively.

Vaccine stability was assessed through Ramachandran plot analysis after refinement. ERRAT analysis of the designed vaccine model yielded an overall quality factor of 96.1702, indicating a high-quality structure suitable for further study (Figure 4a). The Ramachandran plot analysis revealed that 92.9% of the residues fell within the most favored region, while 5.3%, 0.9%, and 0.9% were found in the additionally allowed, generously allowed, and disallowed regions, respectively (Figure 4b). Additionally, structural validation using the ProSA server produced a Z-score of −4.84, further confirming the model’s favorable structural quality (Figure 4c).

### Solubility analysis

Using the Protein-Sol server, the vaccine construct was given a scaled solubility score of 0.504, compared to the population solubility score of 0.45 for *E. coli* proteins (Figure 3). To verify this result, the SOLpro server was used to predict solubility, producing a probability score of 0.819691 for the proposed vaccine construct upon expression. When this value exceeds the threshold of 0.5, the protein is considered soluble according to the SOLpro server.

### Molecular docking analysis

**Figure 5:**
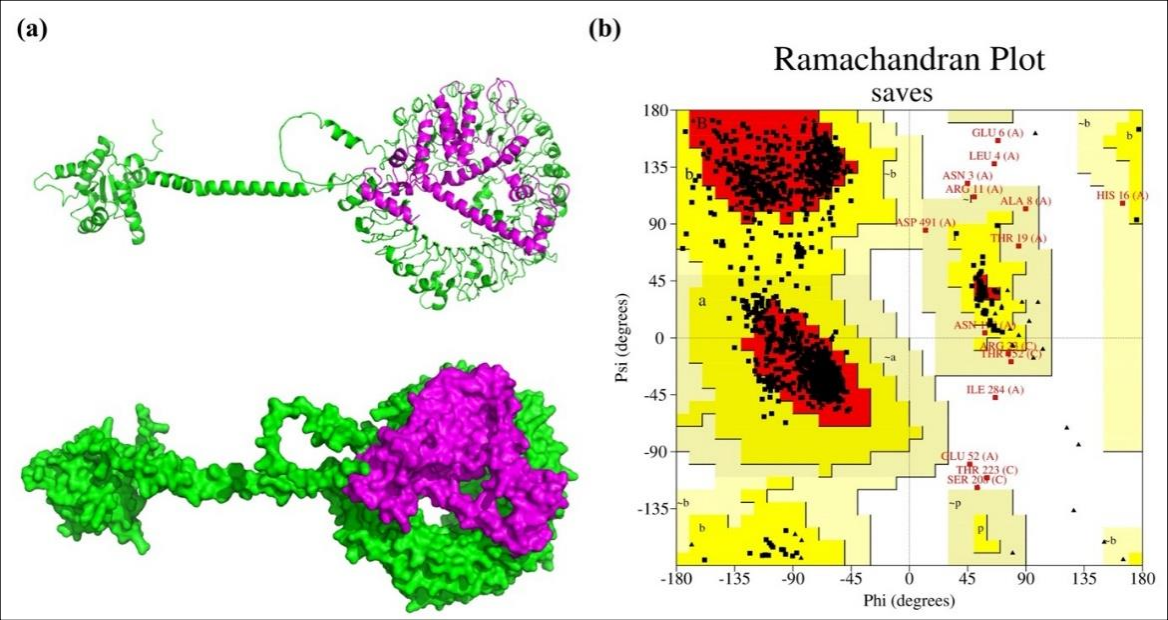
Molecular Docking and Structural Validation of the TLR7–Vaccine Complex. (a) Cartoon representation and surface view of the docking complex (TLR7–designed vaccine), with TLR7 depicted in green and the designed vaccine in magenta. (b) Ramachandran plot analysis of the docking complex, as assessed by the PROCHECK server. TLR7, TLR7–designed vaccine.

**Figure 6:**
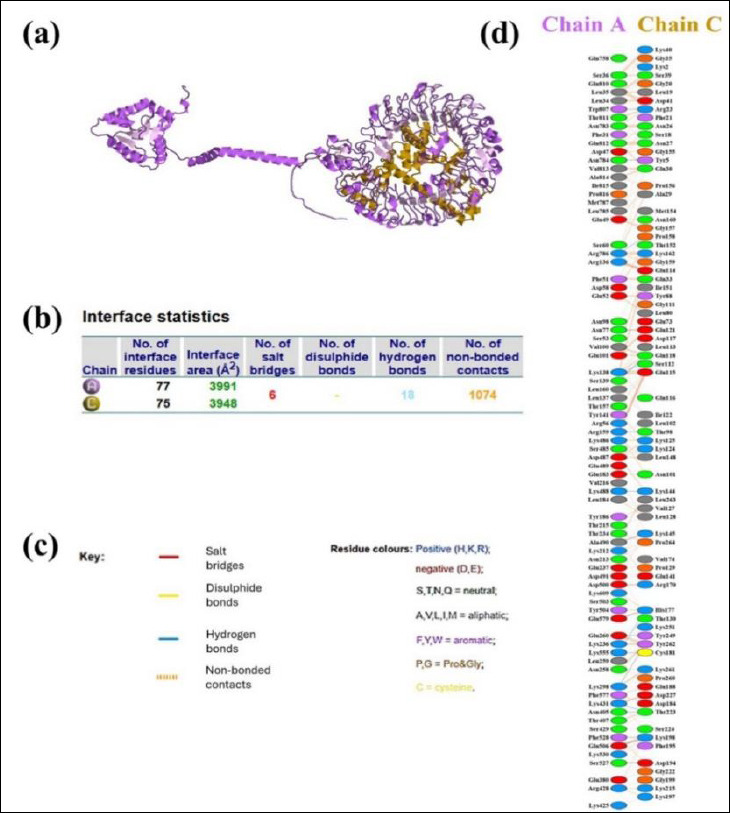
Molecular Interaction Between the Vaccine Construct and Chicken TLR7. (a) Docked complex of chicken TLR7 (purple) and the vaccine construct (gold). (b) Results of the interface statistical analysis. (c) Key residues involved in interactions across the docking interface. (d) Specific interacting residues between the vaccine (chain A) and TLR7 (chain C).

Since antigen–receptor interaction plays a critical role in immune activation, the GRAMM-X docking server was used to model the binding of the vaccine construct to TLR-7. Figure 5 shows the resulting docking complex, generated by the GRAMM-X server and visualized with PyMOL. The quality of the docking complex was further validated by a PROCHECK Ramachandran plot, which indicated that 99.3% of residues were in favored regions, while only 0.7% were in disallowed regions (Figure 5b). These results confirm the structural reliability of the docking complex. As shown in Figure 6, the binding interaction involved the formation of 18 hydrogen bonds, 6 salt bridges, and 1,074 nonbonded contacts. The PRODIGY web server was used to determine the binding affinity of the docked molecules in terms of Gibbs free energy (ΔG) and thermodynamic parameters, including the dissociation constant. These values offer insights into the stability and feasibility of molecular interactions under cellular conditions. The analysis revealed a Gibbs free energy (ΔG) of −33.3 kcal/mol and a dissociation constant of 3.5e−25. The full GRAMM-X docking PDB files are provided in Supplementary Material 1 to ensure transparency and allow independent validation.

### Molecular dynamics simulation

**Figure 7:**
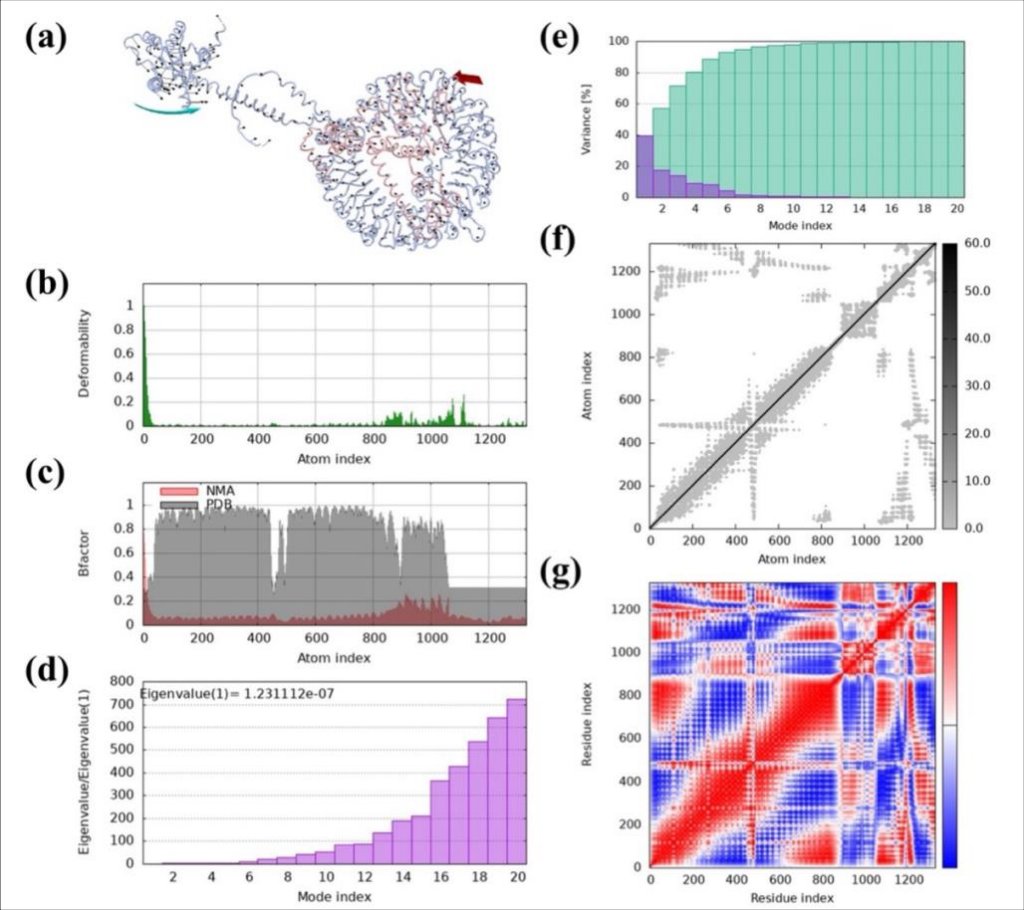
Molecular dynamics (MD) analysis of the vaccine protein complex. (a) Motion direction indicated by red and cyan colors, (b) Stability assessment through low main chain deformability, (c) B-factor/mobility analysis, (d) Eigenvalue demonstrating the protein’s normal mode and motion stiffness, (e) Normal mode variance, (f) Covariance matrix, and (g) Elastic network model showing the stiffer mode of residues. MD, molecular dynamics (MD) analysis.

We performed NMA on the IBV vaccine construct using the iMODS server. Figure 7a depicts the directions of residue movement with arrows, highlighting the protein’s dynamic behavior. Figure 7b shows the deformability of hinge regions along the protein chain, indicating localized flexibility. We derived B-factors from PDB and NMA data, as shown in Figure 7c. The eigenvalue, which reflects the energy required for structural deformation, was used to assess the normal-mode deformability of the vaccine protein. The obtained eigenvalue (1.231112e−07) indicates the molecular motion stiffness, with lower eigenvalues corresponding to greater structural flexibility (Figure 7d). In Figure 7e, normal-mode variance, which is inversely related to the eigenvalue, is displayed with green and purple bars for cumulative and individual variance, respectively. The covariance matrix in Figure 7f maps the relationships between motions: red indicates correlated, white indicates uncorrelated, and blue indicates anticorrelated residue movements. Finally, the elastic network model depicts atom pairs connected by springs, with each spring represented by a dot; the dot’s color intensity reflects stiffness, darker dots indicate higher stiffness, while lighter dots indicate lower stiffness (Figure 7g).

### Immune response simulation and analysis

**Figure 8:**
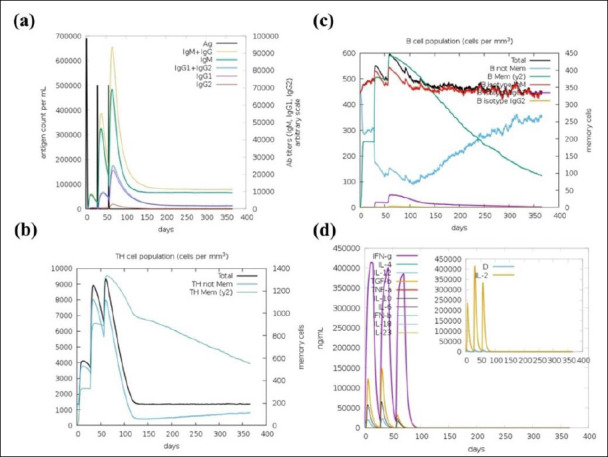
C-ImmSim server immune response simulation of the designed multi-epitope vaccine. (a) Antigen and immunoglobulin response, (b) Helper T cell population dynamics, (c) B lymphocyte population, and (d) Induced interleukins and cytokine levels.

Immune simulations were conducted using the C-ImmSim server to evaluate the immunogenicity of the designed MEV, focusing on key aspects of the immune response. Figure 8a displays the antigen and immunoglobulin (Ig) levels over time. A strong primary immune response occurred after the first dose, with a noticeable drop in antigen levels and a corresponding rise in immunoglobulins, especially IgM, IgG1, and IgG2. Subsequent vaccine doses enhanced this response, indicating robust immunological memory and suggesting the vaccine’s potential to sustain humoral immunity. Figure 8b illustrates the dynamics of helper T-cell populations after each immunization, showing significant expansion and memory development. In Figure 8c, B-cell populations also grew consistently, with a clear increase in memory B cells, supporting long-term antibody production. Figure 8d shows the cytokine and interleukin profiles, which show increased levels of IFN-γ and IL-2 after booster doses, indicating strong T-cell activation and a Th1-biased response. Transient peaks in proinflammatory cytokines, such as Tumor necrosis factor-alpha (TNF-α) and IL-12, reinforced this Th1 bias, while lower levels of Th2-related cytokines, such as IL-4 and IL-10, suggested a balanced response without excessive inflammation. Overall, these results confirm that the vaccine can stimulate both effective humoral and cellular immune responses, with strong memory formation and cytokine signaling.

## DISCUSSION

### Rationale for targeting RdRp in IBV vaccine design

IBV remains a significant threat to the global poultry industry due to its rapid genetic evolution and the limited cross-protection provided by current vaccines [4, 71]. These issues emphasize the urgent need for alternative vaccination strategies, especially immunoinformatics-based MEV designs targeting conserved viral proteins, a strategy that is increasingly gaining attention in veterinary medicine [20, 22, 72]. In this study, we focused on the IBV RdRp, NSP12, a protein essential for viral replication and transcription. RdRp was chosen because of its high sequence conservation (>92.7% globally and up to 98.7% among regional strains, such as those from Iran [73–75]), lack of host homologs, and lower susceptibility to antigenic drift compared to surface proteins, such as the S glycoprotein. This approach signifies a shift toward exploiting conserved NSPs to address IBV antigenic variability and improve T-cell-mediated immunity, complementing antibody responses. Similar strategies targeting RdRp have shown promise in other members of the *Coronaviridae* family [18, 19].

### Advantages of a multi-epitope strategy over conventional IBV vaccines

Traditional live attenuated IBV vaccines carry the risk of reverting to virulence or recombining with field strains [2, 8]. While safer, inactivated vaccines usually produce weak T-cell responses [2]. In contrast, MEVs provide a more targeted and safer alternative by focusing on immunogenic regions without exposing the whole virus. Past in *silico* IBV vaccine research mainly targeted the S protein or multiple structural proteins to increase coverage, but this often made the constructs more complex and increased junctional immunogenicity [20, 76]. By focusing solely on RdRp, the current approach simplifies the vaccine while leveraging its critical role in viral replication to stimulate specific cellular immunity. This type of vaccine could be incorporated into poultry vaccination plans through mass intranasal or oral delivery to boost mucosal and respiratory immunity, complementing existing strategies [71, 74]. However, challenges remain, such as scaling up recombinant expression, improving protein folding, and optimizing adjuvant delivery—especially for avian β-defensin 8 (AvBD8), which may need in ovo or mucosal administration to maximize innate immune response [71, 74].

### Epitope identification and vaccine construct design

Using a comprehensive immunoinformatics framework, we identified multiple B-cell, helper T-cell (HTL), and cytotoxic T-cell (CTL) epitopes within the IBV RdRp sequence. Eight independent prediction servers were employed, and a consensus-based approach was adopted to improve prediction reliability and reduce false positives. Only epitopes predicted by at least three tools were retained. After strict screening for antigenicity (VaxiJen score >0.6), non-allergenicity, non-toxicity, and IFN-γ induction potential (for HTL epitopes), five BCL epitopes, three HTL epitopes, and three primary CTL epitopes were selected for vaccine design (Tables 1–3). Notably, five additional CTL epitopes were identified within the selected B-cell and HTL sequences (Table 4), suggesting potential multifunctional immunological hotspots capable of eliciting coordinated humoral and cellular immune responses.

These epitopes formed the foundation of the final MEV construct (Figure 2a), assembled using carefully chosen linkers. AAY linkers were included to improve proteasomal processing and CTL epitope presentation, GPGPG linkers offered structural flexibility and maintained domain independence, and KK linkers enhanced solubility and aided lysosomal processing for MHC class II presentation [49]. To further boost immunogenicity, avian β-defensin 8 was added as an N-terminal adjuvant through an EAAAK linker. β-defensins are known to be immunomodulators and TLR agonists, stimulating proinflammatory cytokines and chemokines in chicken immune cells, likely via MAPK signaling pathways [77].

### Structural stability, solubility, and innate immune engagement

The resulting vaccine construct (Figure 2b) demonstrated favorable *in silico* physicochemical and structural properties. Structural modeling, refinement, and validation confirmed a stable, hydrophilic, and thermotolerant protein with strong stereochemical integrity, as shown by a high percentage of residues in favored Ramachandran regions (92.9%), a high ERRAT quality score (96.1702), and a ProSA Z-score (−4.84) within the range of native proteins. The predicted instability index (25.74) and solubility score (0.504, above the *E. coli* average of 0.45; Figure 3b) further indicate suitability for scalable recombinant production and formulation.

Molecular docking analysis showed a strong and specific interaction between the vaccine construct and TLR7, characterized by very low Gibbs free energy and high binding affinity, supported by 18 hydrogen bonds, six salt bridges, and 1,074 non-bonded contacts (Figure 5). These results suggest effective engagement of innate immune receptors, which is essential for triggering downstream adaptive immune responses against ssRNA viruses such as IBV.

### Dynamic stability and immune response simulation

NMA further confirmed the structural strength and flexibility of the vaccine construct, which are crucial for effective antigen processing and presentation (Figure 7). The low eigenvalue (1.231112e−07) showed good deformability, especially at hinge regions, aiding immune recognition. In *silico* immune simulations predicted a strong and lasting immune response, including efficient antigen clearance and robust humoral immunity dominated by IgG, with peaks in IgM, IgG1, and IgG2 after each dose (Figure 8). Expansion of memory B cells and T helper cells indicated the potential for long-term immunological protection.

Cytokine profiling revealed a predominant IFN-γ response, consistent with Th1-mediated antiviral immunity, along with brief increases in IL-2, TNF-α, and IL-6, which are essential for T-cell growth and immune regulation. Overall, these results suggest that the designed vaccine can induce balanced and long-lasting humoral and cellular immune responses.

### Implications for cross-protective immunity and vaccine design

The results support RdRp as a promising vaccine target capable of eliciting comprehensive immune responses that interfere with viral replication. RdRp-derived epitopes demonstrated strong TLR7 binding and Th1-dominated immune profiles with elevated IFN-γ and IgG production (Figures 6 and 8), suggesting the potential to disrupt RdRp-mediated RNA synthesis in infected respiratory and renal tissues. In avian models, such immune responses are linked to reduced viral load, mitigation of nephropathogenesis, and decreased transmission, as RdRp-specific CTL epitopes eliminate infected cells while antibodies interfere with polymerase function [78–80]. These findings align with evidence from related coronaviruses, where RdRp targeting has been connected to durable cross-protective immunity against emerging variants [18, 19, 21, 81].

By focusing solely on RdRp, this study presents a streamlined vaccine construct with high stability and solubility, avoiding issues like structural instability or unintended junctional immunogenicity that may occur in multi-protein designs [76, 82]. Although adding additional conserved antigens, such as NSP13 or conserved spike domains, could theoretically expand immune coverage, such approaches might reduce scalability and manufacturing feasibility. As a proof-of-concept, the current single-target strategy shows strong in *silico* immunogenicity and provides a solid foundation for future multi-protein vaccine designs that combine RdRp with complementary antigens to achieve synergistic protection.

### Limitations and future perspectives

Despite promising in *silico* results, several limitations need to be acknowledged. Using human MHC alleles as proxies for chicken MHC molecules, though supported by prior studies, may not fully reflect avian-specific peptide-binding preferences and structural differences [38]. Additionally, computational predictions cannot fully capture the complexity of in *vivo* immune responses, and IBV field strain diversity may affect vaccine coverage despite the high conservation of RdRp [35–40]. Population coverage analysis could not be conducted due to the lack of validated avian MHC datasets and allele frequency data.

Future studies should focus on experimental validation, including *in vitro* assays with chicken peripheral blood mononuclear cells, enzyme-linked immunosorbent assays, and *in vivo* vaccination–challenge trials against various IBV strains from different regions. These studies will be crucial for confirming immunogenicity, safety, and cross-protective efficacy. Overall, this research offers a strong immunoinformatics-based framework for developing RdRp-based MEVs and provides a promising basis for next-generation IBV vaccines and broader strategies against rapidly evolving avian pathogens.

## CONCLUSION

This study presents an immunoinformatics-driven design of a novel MEV targeting the RdRp of IBV, a highly conserved and replication-essential NSP. Comprehensive in *silico* analyses demonstrated that the selected RdRp sequence exhibits strong conservation across IBV isolates (>92.7%), supporting its suitability as a broadly representative vaccine target. The rational epitope selection strategy identified high-confidence B-cell, helper T-cell (HTL), and cytotoxic T-cell (CTL) epitopes with favorable antigenicity, non-allergenicity, non-toxicity, and IFN-γ induction potential. The final vaccine construct showed excellent physicochemical stability, solubility, and structural integrity, with a low instability index, favorable Ramachandran statistics, and high ERRAT and ProSA scores. Molecular docking revealed strong and specific interaction with chicken TLR7, while molecular dynamics and immune simulations predicted robust Th1-skewed immune responses characterized by elevated IFN-γ production, IgG-dominated humoral immunity, and the formation of immune memory.

From a practical standpoint, the proposed RdRp-based MEV offers several advantages over traditional IBV vaccines. By targeting a conserved NSP rather than highly variable surface antigens, this approach has the potential to overcome serotype-specific limitations and provide broader, more durable protection against diverse IBV strains. The inclusion of avian β-defensin 8 as an endogenous adjuvant further enhances innate immune activation relevant to ssRNA viruses. Additionally, the predicted solubility and stability of the construct suggest feasibility for recombinant production and downstream formulation, making it suitable for scalable poultry vaccination programs and potential integration with existing immunization strategies.

A key strength of this study is its comprehensive and rigorous immunoinformatics pipeline, which combines consensus-based epitope prediction, thorough safety and antigenicity screening, structural modeling and validation, receptor-binding analysis, molecular dynamics, and immune response simulation. Focusing solely on RdRp allowed for a streamlined vaccine design, reducing construct complexity and the risk of unintended junctional immunogenicity, while enhancing immunological relevance.

In conclusion, this proof-of-concept study offers strong computational evidence that RdRp is a promising vaccine target for IBV and that a carefully designed multi-epitope construct can stimulate coordinated innate, humoral, and cellular immune responses. Although experimental validation is necessary to confirm immunogenicity, safety, and protective efficacy in *vivo*, the findings provide a solid foundation for next-generation IBV vaccine development and present a transferable framework for tackling other rapidly evolving avian viral pathogens.

## DATA AVAILABILITY

The supplementary data can be made available from the corresponding author upon request.

## AUTHORS’ CONTRIBUTIONS

GNB, RR, FF, SG, MHM, and BAK: Conceptualized and designed the study. MHM, SG, and RR: Data collection. GNB, MHM, SG, RR, BAK, and FF: Conducted literature review, data analysis, and interpretation. MHM, SG, RR, and GNB: Drafted the manuscript. SG, MHM, and GNB: Revised the manuscript. Supervision: GNB. All authors have read and approved the final version of the manuscript.
